# Cost of illness of scrub typhus in South India – a population-based, mixed-methods study

**DOI:** 10.1371/journal.pntd.0013960

**Published:** 2026-02-02

**Authors:** Carol Devamani, Adam Biran, Koya Ariyoshi, K.R. John, Ian Ross, Yoshinao Kubo, Daniel Chandramohan, Kundavaram Paul Prabhakar Abhilash, Wolf-Peter Schmidt

**Affiliations:** 1 Department of Child Health-3, Christian Medical College Vellore, Vellore, India; 2 Population Health Sciences Institute, Newcastle University, Newcastle upon Tyne, United Kingdom; 3 Department of Infectious Disease Epidemiology, Institute of Tropical Medicine, Nagasaki University, Nagasaki, Japan; 4 Department of Community Medicine, AIMSR, Chittoor, India; 5 Department of Health Services Research and Policy, London School of Hygiene and Tropical Medicine, London, United Kingdom; 6 Department of Clinical Medicine, Institute of Tropical Medicine, Nagasaki University, Nagasaki, Japan; 7 Department of Disease Control, London School of Hygiene and Tropical Medicine, London, United Kingdom; 8 Department of Emergency Medicine, Christian Medical College Vellore, Vellore, India; Yale University School of Medicine, UNITED STATES OF AMERICA

## Abstract

**Background:**

Scrub typhus is a potentially life-threatening acute febrile illness found in many parts of Asia. This study aimed to estimate the cost of illness among scrub typhus cases in Tamil Nadu, South India and explore treatment-seeking behaviour.

**Methods/Principal Findings:**

Cases were enrolled from a population-based cohort study on scrub typhus in 32,279 individuals living in rural villages. Data on direct and indirect costs were collected using structured questionnaires from 311 scrub typhus cases of which 26 were severe. Thirteen cases with severe infections (or their relatives) underwent in-depth interviews to understand treatment-seeking pathways.

The mean age of cases was 41.1 years, and 64% were female. The average monthly household income was USD 236 (standard deviation, SD 155). The average number of workdays missed in severe infection was 18 days per episode (SD 22.2) compared to 4 days (SD 11.6) in non-severe illness. The mean overall cost of illness was USD 189 (SD 495), disaggregating to USD 1,321 (SD 1045) for severe cases and USD 86 (SD 209) for non-severe. In both severe and non-severe cases, hospital admission was common (69/311) and was associated with a cost increase of over USD 400. Costs were almost twice as high in males compared to females. Catastrophic health expenditure exceeding 25% of annual income occurred in 10% of cases. Treatment by traditional healers, untrained practitioners, pharmacies and local clinics was sought even for mild fever of short duration. In-depth interviews revealed that patients preferred to have a one-off treatment enabling them to return to daily routines with little demand for fever diagnostics. There was demand for higher level of care and diagnostic procedures only when symptoms became severe or the case was a child or a pregnant woman.

**Conclusions/Significance:**

Hospitalisation, common in both severe and non-severe patients, was the driving factor for high costs. Early case recognition may reduce hospitalisations and health expenditure in highly endemic settings.

## Authors summary

Scrub typhus is an important cause of fever in many Asian countries, including China, India, Vietnam and Japan. It is caused by the intra-cellular bacteria *Orientia (mostly O. tsutsugamushi)* and transmitted to humans by trombiculid mite larvae (“chiggers”). Scrub typhus is potentially life-threatening, but treatable with cheap antibiotics such as doxycycline and azithromycin. In this study, the cost of illness was determined in 311 cases identified as part of a cohort study on the incidence of scrub typhus in a rural setting in South India. In-depth interviews were conducted to determine treatment seeking behaviour. The mean overall cost of illness per case was USD 189, disaggregating to USD 1,321 for severe cases and USD 86 for non-severe cases. Hospitalisations were found to be the driving factor for high costs in both mild and severe cases, often leading to health expenditures exceeding the average monthly household income of USD 236.

## Introduction

Scrub typhus is an acute febrile illness caused by the bacteria of the genus *Orientia* (family: *Rickettsiaceae* [1]) and transmitted by the larval stage (chigger) of trombiculid mites [1,2]. Scrub typhus is endemic to tropical and sub-tropical East Asia, South Asia and South East Asia, but has also been found in Chile [3], the Middle East and East Africa [4].

Hospital-based studies in the South Asian region have suggested scrub typhus as a leading cause of severe acute undifferentiated febrile illnesses [5,6], and is marked by acute respiratory distress syndrome, meningo-encephalitis, shock, renal failure and other complications [5,7–10]. Severe infections can be prevented by early use of antibiotics such as doxycycline and azithromycin [11]. Since treatment of scrub typhus often involves intensive care, the cost of infection at a system and individual level is of particular interest, but under-researched. A recent single-centre study in a private tertiary hospital in the South Indian state of Tamil Nadu estimated the median direct medical costs of hospitalized patients with scrub typhus at USD 490 (interquartile range 304–860) [12].

The health system in India comprises private and public health care providers, both offering primary, secondary and tertiary care. Public providers charge no fees for consultations. Private providers include both qualified (Bachelor of Medicine - Bachelor of Surgery (MBBS) or higher) and unqualified practitioners. Most hospitals require relatives to be present throughout the patient’s stay and to provide food for the patient, assist in personal hygiene and pay bills as they arise. The majority of individuals in India lack health insurance [13]. Therefore, out of pocket health expenditures are significant, with medicines accounting for nearly 72% of the out-of-pocket expenditures in one nationally-representative study [14]. Selvaraj and colleagues suggested that every year, 55 million Indians fall below the poverty line due to healthcare costs [13].

We conducted a study to determine the direct and indirect costs of scrub typhus infection, and estimate the risk of catastrophic health expenditure, considering treatment seeking pathways at different levels of disease severity. Given the fragmented healthcare system in India, it seemed likely that costs would be strongly influenced by household choices with respect to healthcare providers. Therefore, to complement the quantitative analysis, a qualitative study was conducted in a subset of cases to better understand treatment-seeking behaviour.

## Methods

### Ethics statement

The study was approved by the Institutional Review Board of Christian Medical College Vellore(Ref: 11726) and London School of Hygiene and Tropical Medicine’s Research Ethics Committee (Ref: 16573). The overall cohort study is registered at clinicaltrials.gov (NCT04506944). Written consent was obtained from all adult participants. Written or verbal assent was obtained from minors, alongside written consent from their parent/guardian.

### Study population

Cases for the present study were enrolled from a population-based cohort study estimating the incidence of scrub typhus in a population of 32,279 individuals from 7619 households living in 37 villages in two districts (Vellore and Ranipet) in Tamil Nadu, South India over a 2-year period from February 2020 [15]. Villages were dominated by agriculture, with rice, peanuts, banana, taro, sugarcane, coconut, pulses and turmeric being major crops. Animal husbandry included cattle, goats and poultry. The incidence of symptomatic scrub typhus infection in the study population was estimated to be 6.0 cases per 1000 person-years (95% CI 0.4 to 0.8).

### Study design

This was a mixed-method, cross-sectional study. Costs were estimated from cases occurring during the follow-up period of the cohort study, based on participant or caregiver recall in a household survey.

### Recruitment and sampling

During the two years of follow-up of the main cohort study, households were visited every 6–8 weeks. Those who had a history of fever from the time of the last visit or during the previous 2 months (whichever was shorter) were asked to give a venous blood sample. Thirteen rounds of household visits were conducted over the 2-year follow-up period. However, due to COVID, two rounds were cancelled at the time of government-enforced movement restrictions. Using our case definition for scrub typhus (see below), 328 cases of scrub typhus were identified, of which 29 cases were classified as ‘severe’, defined as having respiratory failure, encephalitis, miscarriage, kidney failure and/or shock [15]. All cases were eligible for enrolment in the present study and invited to take part in a structured questionnaire survey. For the qualitative study, we recruited a random selection of 13 from the list of 29 severe cases, stratified by age of case (<20 years, 20–49 years and 50 years or higher) and sex, to ensure broad representation of cases. The qualitative study ended after interviewing 13 cases, as no additional themes were emerging.

### Data collection

Quantitative data were collected through a structured questionnaire survey administered verbally by four trained field workers. Survey interviews took place within three months of the case being identified in the cohort. If possible, the case was interviewed. If the case had died (due to scrub typhus or other causes), the interview was with family members. For cases younger than 18 years, the primary care giver (individual who cares for the child on most days) was interviewed. Data included clinical characteristics, treatment, illness-associated direct and indirect costs, household income, and caste (classified as general, other backward classes/most backward class (OBC/MBC) and scheduled caste/tribe (SC/ST). The terms OBC/MBC and SC/ST generally describe historically disadvantaged groups eligible for certain social benefits such as reservation for government positions and access to higher education. The category SC/ST overlaps with the term *Dalit*, used to describe groups that were historically considered “untouchables”.

Qualitative data were collected through in-depth interviews by CD, one of the investigators. Each interview began by working through the topic guide with a single participant, either the case, a close family member (if the case was not available) or the primary caregiver (if the case was a child). Once this was completed, other family members present were invited to participate to help fill in gaps and allow for clarifications. The main topics discussed included the treatment pathway from the start of symptoms to the eventual treatment of fever, the choice of healthcare providers and reasons for the choice. Interviews were assisted with participatory tools, including stick figures and photos of various healthcare providers to engage the participants in recalling the treatment pathway [16].

### Analysis

Cost data were analyzed as direct and indirect costs. Direct costs included out-of-pocket payments categorised as “medical” and “non-medical”. Medical costs pertained to the treatment costs such as registration, consultations, blood tests, x-rays and medications; non-medical costs included food and travel related to the illness; indirect costs included the loss of income to the patient and accompanying family for the consultation or admission to a hospital.

Our case definitions for non-severe and severe scrub typhus have been published [15]. In brief, scrub typhus was diagnosed based on a positive IgM Enzyme Linked Immunosorbent Assay (ELISA) or a positive polymerase chain reaction (PCR). Severe scrub typhus was defined as the presence of organ involvement, in particular respiratory failure, encephalitis, kidney failure, shock or miscarriage. Hospitalisation was defined as at least one night of admission in a hospital [15]. Other care levels were categorized as hospital outpatient department, private clinic, pharmacy and no treatment.

The daily income lost was based on the monthly income divided by the number of workdays. The average working day was calculated assuming an 8-hour working day. Since most cases were daily wage workers, lost work equaled loss of income. The loss in income for the carer of a case was based on the number of workdays lost to caring for the case. Mothers of patients who were children (<18 years), were accounted for with a loss of wage during the days of illness for the child. The time spent by people (as case or carer) who didn’t have regular paid work was valued at 50% of the average daily wage following reference case guidelines [17]. The average daily wage was determined based on information from the qualitative study and set to Indian Rupees (INR) 600 (USD 7.7) for men, and INR 500 (USD 6.4) for women. Loss of education time in school children and college students was valued at half the adult value (i.e., 25% of wages). There is considerable uncertainty about methods in this area, but it is common practice that children’s time is valued lower than adults’ and the teenage wage rate can generally be assumed to be lower [18]. To understand how these assumptions influence the overall estimates we conducted sensitivity analyses, assuming the unpaid worker and students to have either no income loss or 100% income loss of average daily wage.

We used two different thresholds to define catastrophic health spending, at 10% and 25% of annual household income [19]. Annual household income was used as a proxy for household expenditure. The numerator for this calculation was total out-of-pocket expenditure for the illness [20]. Costs were compared for severe versus non-severe cases and hospitalized versus ambulatory cases. Clinical characteristics were compared between severe versus non-severe cases using t-tests (continuous variables), and chi-square tests (categorical variables). The highest level of care was defined as the highest level amongst all the treatments/facilities visited for care, i.e., hospital admission being the highest care level followed by hospital outpatient department, private clinic or pharmacy and finally no treatment.

Univariable and multivariable generalized linear models (log link, gamma family) [21] were used to examine associations between total costs per patient and demographic, economic and clinical factors including age, sex, farming, level of education, severity of illness and hospitalisation. Differences were calculated as marginal effects. All costs were reported in US dollars (USD 1 = INR 78.1 in July 2022).

Qualitative data were analyzed thematically [16,22]. In-depth interviews were audio-recorded, anonymized, transcribed verbatim in Tamil, translated into English and checked by native speakers for accuracy of the translation.

Transcripts from two pilot interviews were coded by two of the authors (CD and AB) and codes were discussed across the wider team and a provisional coding framework agreed, which was applied to the remaining transcripts by CD after familiarization with the data. Coding of the full dataset was initially based on the coding framework but allowed for further codes to be included as the analysis progressed. Further codes proposed were discussed with the team and, if considered useful, were retrospectively applied to earlier transcripts [23]. The Model of Pathways to Treatment (MPT), which proposes a generic ‘pathway’ from the experience of symptoms to receipt of treatment [24] was used as a framework to guide the qualitative study analysis.

## Results

### Quantitative study

Questionnaire data for the present study was collected for 311 of 328 scrub typhus cases (95%), including 26 out of 29 severe cases (89%). One individual had two episodes in the study period (2 years). The remaining cases (or their surviving family members) did not consent for an interview (n = 14) or were unavailable after several attempts were made for the interview (n = 3).

The mean age of cases was 41.1 years (range 2 – 90 years) and 199 (64%) were female (Table 1). The mean age of non-respondents was 47.0 years (SD 21.5), with 10 out of 17 being female (59%). Most cases belonged to the MBC/OBC caste categories. Most of the cases were educated to the primary level. Fifty cases had no formal education. Two-thirds of cases were doing some form of farming, mainly as full-time farmers. Part-time farming was practiced by about a quarter of the cases. Most of the households had a low income, with an overall mean household monthly income at INR 18,432 (USD 236).

**Table 1:** Sociodemographic characteristics of study participants.

Variable	n (%)
Age group, years (N = 311)	
< 18	52 (16.7)
18 – 35	65 (20.9)
36 – 55	109 (35.1)
≥ 56	85 (27.3)
	
Sex (N = 311)	
Male	112 (36.0)
Female	199 (64.0)
	
Caste (N = 311)	
SC/ST	147 (47.3)
MBC/OBC	161 (52.8)
General caste	3 (1.0)
	
Education (N = 311)	
None	50 (16.1)
Primary	118 (37.9)
Secondary (6–10^th^)	97 (31.2)
Higher secondary (11^th^/12^th^)	22 (7.1)
Diploma/University	24 (7.7)
	
Farming (N = 311)	
Full time	132 (42.4)
Part time	76 (24.4)
None	103 (33.1)
	
Household’s monthly income, USD (N = 311)	
0	–
12 - 100	54 (17.4)
101 - 300	199 (64.0)
301 – 600	52 (16.7)
601 - 900	6 (1.9)

Among severe cases, the respiratory system was the most common involvement, followed by shock, renal failure, central nervous system involvement, myocarditis and miscarriage (Table 2). There were 4 deaths. All but one of the severe cases were admitted to a hospital, while 15% of non-severe cases were admitted. Severe cases were on average admitted for 3 days more than non-severe cases. The average number of facilities visited in severe cases (2.8) was almost twice as high as in non-severe cases. Overall, the most common highest level of care was a private clinic or pharmacy, followed by similar proportions in hospital outpatient treatment and hospital admission (Table 2). Only one (non-severe) case had no treatment at all. Private hospital outpatient services and inpatient services were used more often than government hospitals in severe cases, but not in non-severe cases.

**Table 2:** Clinical related characteristics of study participants.

	Overall (N = 311)	Non-severe (N = 285)	Severe (N = 26)	p-value
				
Admitted (%)	69/311 (22.2)	44/285 (15.4)	25/26 (96.2)	<0.001
				
Mean Days Admitted (SD)*	5.7 (3.5)	4.5 (2.5)	7.7 (4.0)	<0.001
Range (days)	1-17	1-12	1-17	
Mean days of fever, (SD)	5.8 (4.4)	5.5 (4.3)	9.3 (4.2)	<0.001
Range (days)	1-25	1 - 25	3 - 17	
				
Complications (%)				
Lung involvement			18 (69%)	
Shock			10 (38%)	
Renal Failure			5 (19%)	
CNS			3 (12%)	
Myocarditis			2 (8%)	
Miscarriage			2 (8%)	
Death			4 (15%)	
				
Average number of treatment facilities visited (SD)	1.7 (1.0)	1.6 (0.9)	2.8 (1.3)	<0.001
Proportion of Various treatment facilities used (%)				
No treatment	1/311 (0.3)	1/285 (0.4)	0	0.762
Traditional- Healer, Ayurvedic, Homeopathy	18/311 (5.8)	17/285 (6.0)	1/26 (3.9)	0.658
Pharmacy/Shop	88/311 (28.3)	85/285 (29.8)	3/26 (11.5)	0.048
Local practice/clinic	219/311 (70.4)	198/285 (69.5)	21/26 (80.1)	0.227
Hospital Outpatient Department (Government)	71/311 (22.8)	64/285 (22.5)	7/26 (26.9)	0.603
Hospital Outpatient Department (Private)	71/311 (22.8)	60/285 (21.1)	11/26 (42.3)	0.013
Hospital inpatient Department (Government)	25/311 (8.0)	18/285 (6.3)	7/26 (26.9)	0.000
Hospital Inpatient Department (Private)	46/311 (14.8)	26/285 (9.1)	20/26 (76.9)	0.000
				
Highest Level of Care				0.000
No treatment	1/311 (0.3)	1/285 (0.4)	0	
Private clinic/Pharmacy	170/311(54.7)	169/285 (59.3)	1/26 (3.9)	
Hospital Outpatient Department.	71/311 (22.8)	71/285 (24.9)	0	
Hospital Admission	69/311 (22.2)	44/285 (15.4)	25/26 (96.2)	

*In those admitted.

A total of 26 severe cases incurred a mean direct medical cost of USD 1,073, while the 285 non-severe cases were USD 51 (Table 3). The mean direct non-medical costs for severe cases were relatively low (USD 52) and even lower for non-severe cases (USD 5). Among indirect costs, carers for the patient with severe illness incurred a higher loss of income than the case itself, while the opposite was true for non-severe cases (Table 3). The average number of workdays missed in severe cases was 18 days (SD 22.2) compared to 4 days in non-severe cases (SD 11.6, difference 14, 95% CI -19.2 to -8.8).

**Table 3:** Cost of illness (USD) of study participants.

		Non-Severe vs. Severe			Ambulatory vs. Hospitalized		
	TOTAL (n = 311)	NON-SEVERE (n = 285)	SEVERE (n = 26)	Difference (95% CI)	AMBULATORY (n = 242)	HOSPITALISED (n = 69)	Difference (95% CI)
	**Mean (SD)**	**Mean (SD)**	**Mean** **(SD)**		**Mean** **(SD)**	**Mean** **(SD)**	
OVERALL TOTAL	189 (495)	86 (209)	1321 (1045)	-1235 (-1379, -1090)	36 (82)	724 (849)	-688 (-797, -579)
DIRECT COSTS							
Direct Total	146 (441)	57 (188)	1125 (963)	-1068 (-1200, -936)	13 (41)	612 (774)	-599 (-697, - 501)
Medical	137 (429)	51 (180)	1073 (957)	1022 (-1152, -892)	11 (38)	578 (761)	567 (-470, -663)
Non-medical	9 (22)	5 (13)	52 (46)	-46 (-39, -54)	2 (5)	34 (36)	-32 (-27, -37)
INDIRECT COSTS							
Indirect Total	43 (110)	29 (64)	196 (276)	-167 (-207, -127)	23 (60)	112 (188)	-89 (-117, -61)
Loss of income							
Patient	27 (74)	22 (61)	80 (154)	-58 (-88, -29)	19 (59)	54 (109)	-35 (-54, -15)
Carer	16 (74)	7 (17)	116 (233)	-109 (-136, -81)	4 (13)	58 (149)	-54 (-73, -35)
CATASTROPHIC HEALTH EXPENDITURE							
Health Expenditure Percentage (%)	**TOTAL** **(n = 311)**	**NON-SEVERE** **(n = 285)**	**SEVERE** **(n = 26)**	**p-value**	**AMBULATORY** **(n = 242)**	**HOSPITALISED** **(n = 69)**	**p-value**
>10% (%)	47 (15)	25 (9)	22 (85)	<0.001	9 (4)	38 (55)	<0.001
>25% (%)	30 (10)	11 (4)	19 (73)	<0.001	3 (1)	27 (39)	<0.001

The mean overall cost of illness per case was USD 189 (95% CI: 134, 244; median: USD 20.6; interquartile range: USD 9.7 – 77.7), with a mean overall direct cost of USD 146 (95% CI:96.8, 195.3; median: USD 6.4; interquartile range: USD 2.6 – 30.7) and an overall mean indirect cost of USD 43 (95% CI: 30.6, 55.0; median: USD 12.8; interquartile range: USD 6.4 – 32.0) This, however, varied considerably by severity, with a mean of USD 1,321 for severe cases and USD 86 for non-severe cases. The mean overall cost per patient for severe cases was 15 times higher than for non-severe cases (difference of USD 1235, 95% CI 1090, 1379). Hospitalisation was common, even among non-severe cases, and was the key factor in driving costs (Table 3). In hospitalised patients, the loss of income for the patient and carer were similar. Fifteen percent of cases experienced a catastrophic health expenditure exceeding 10% of their annual household income, while 10% of the case’s expenditure exceeded the 25% threshold (Table 3). In severe cases, catastrophic health expenditure occurred in about 85% or 73% of cases at the thresholds exceeding 10% or 25% of annual income, respectively. For non-severe cases it was 9% and 4%. In hospitalised cases, catastrophic health expenditures exceeding the 10% and 25% thresholds occurred in 55% and 39%.

A sensitivity analysis was done assuming the unpaid worker and students to have either no income loss or 100% income loss of average daily wage. With no income loss, the mean overall indirect cost was found to be USD 35 (SD 109) and mean overall total cost to be USD 181 (SD 493). At the 100% income loss level, the mean overall indirect cost was found to be USD 52 (SD 113) and mean overall total cost to be USD 198 (SD 500).

In univariable analysis, total cost was found to be twice as high in males as compared to females (Table 4), and a substantial difference remained after adjusting for covariates. Similarly, direct costs were higher in males than females (USD 222, vs USD 103, difference -119, 95% CI -17.6, -221.4). Differences observed in costs between different caste groups and by farming as an occupation were strongly reduced in the multivariable analysis. Similarly, those having a higher education or a higher household income had higher costs as compared to those who had no education or lower income, but adjusting for age, sex and disease severity led to a pronounced reduction in these associations. The higher costs incurred by severe compared to non-severe cases were strongly reduced by adjusting for age, sex, income and education. Similarly, the difference in costs of being hospitalised was reduced when adjusting for age, sex, income, education and severity, but was still USD 443 higher than for ambulatory cases (Table 4).

**Table 4:** Univariate and Multivariable Linear Regression Analysis – (USD).

Variables	n	Mean (SD)	Difference	95% CI	Adjusted difference	95% CI	Adjusted for
**Age**							Sex, education, severity
< 20	58	189.8 (379.1)	Ref		Ref		
20 to <40	84	231.6 (662.9)	41.8	-132.8, 216.5	-130.1	-352.8, 92.6	
40 to <60	100	144.8 (334.4)	-45.0	-186.8, 96.8	-2.0	-242.6, 238.5	
≥ 60	69	199.7 (541.4)	9.9	-160.5, 180.3	-152.3	-385.9, 81.2	
**Age (continuous per 10 years increase)**			-4.9	-31.1, 21.2	-14.2	-45.5, 17.1	
**Sex**							Age, education, severity, farming
Male	112	281.1 (653.5)	Ref		Ref		
Female	199	136.9 (370.1)	-144.3	-287.0, -1.6	-117.1	-283.0, 48.8	
							
**Caste**							Age, sex, education, severity
Scheduled Caste/tribe	147	135.6 (356.9)	Ref		Ref		
Most backward class (MBC)/Other backward class (OBC)	161	235.2 (588.1)	99.5	-10.6, 209.7	9.6	-101.0, 120.2	
General caste	3	309.0 (257.2)	173.4	-736.3, 1083.0	-8.8	-552.6, 534.9	
**Farming**							Age, sex, education, severity
None	103	177.2 (401.9)	Ref		Ref		
Part time	76	304.4 (721.3)	127.3	-70.2, 324.8	9.3	-159.1, 177.7	
Full time	132	131.3 (381.6)	-45.8	-151.4, 59.7	-9.5	-137.6, 118.6	
**Education**							Age, sex, severity
None	50	137.9 (350.7)	Ref		Ref		
Primary	118	146.0 (374.0)	8.1	-107.8, 124.1	-22.0	-244.5, 200.5	
Secondary (6–10^th^)	97	219.9 (539.6)	82.0	-63.1, 227.2	-78.2	-325.3, 168.9	
Higher secondary (11^th^/12^th^)	22	49.7 (137.7)	-88.1	-196.8, 20.5	-167.9	-430.7, 95.0	
Diploma/University	24	507.6 (977.9)	369.7	-146.4, 885.9	-100.2	-407.1, 206.8	
**Education (years of schooling)**			30.3	-0.7, 61.2	-24.4	-68.3, 19.5	
**Household income per 10 USD**			5.1	0.2, 10.0	3.0	-1.3, 7.2	Age, sex, education, severity
**Severe**							Age, sex, education, HH income
No	285	85.5 (208.8)	Ref		Ref		
Yes	26	1321.0 (1044.7)	1235.4	41.5, 2429.4	533.1	35.8, 1030.4	
**Hospitalised**							Age, sex, education, severity, HH income
No	242	36.2 (82.3)	Ref		Ref		
Yes	69	724.2 (848.8)	688.0	332.2, 1043.8	442.7	193.6, 691.7	

### Qualitative study

The qualitative study collected data on thirteen cases (8 females and 5 males, median age 50 years, range 5–68). In ten cases, the case itself was the primary respondent, while in the remaining three, the primary respondent was the mother (child 5 years of age), the spouse or the father. We organized our data around 3 overarching themes derived from MPT: ‘Healthcare service factors’, ‘Disease factors’ and ‘Patient factors’ (Table 5). These are outlined below with illustrative quotes.

**Table 5:** Summary of key themes from the qualitative study.

Overarching Themes (MPT Factors)	Topics	Description
Health Service		
	Costs	Direct financial cost associated with treatment through a particular provider
	Convenience	Ease of accommodating treatment within other commitments (paid and unpaid work)
	Likelihood of a good service	Perceived likelihood of being treated attentively based on experience, recommendations or personal connections
Disease		
	Perceived severity	Additional symptoms, loss of appetite
	Perceived cause	Common/ familiar or unknown illness
Patient		
	Biological characteristics	Age, sex and pregnancy status
	Social characteristics	Availability of support for travel, hospitalisation and/or to attend to domestic responsibilities

#### Health services factors.

Participants were aware of a range of healthcare services including traditional healers, untrained practitioners, small clinics, pharmacies, secondary hospitals (private and public) and tertiary level hospitals (private and public). The use of a traditional healer was nearly universal in our sample, usually at the start of the treatment-seeking process.

“*Evil spirits are roaming in the open and they may suddenly come onto us, so we need to always do mantras.” (ID#1, M, 58yrs)*

Seeking the help of a traditional healer was often done in parallel with the use of allopathic medicine and did not seem to delay treatment seeking elsewhere:

*“Evil spirits can come onto us, especially when pregnant, so we must be more careful. [In the case of an illness] we get mantras 2-3 times a day for a few days, along with going to hospitals.” (ID#8, F, 19yrs)*

Participant responses suggested three broad dimensions on which healthcare providers were assessed prior to selection; the costs of using the service, convenience of the service and the likelihood of receiving a good service. Participants’ consideration of the costs of using a service comprised the direct costs of treatment, travel costs and opportunity costs of missed work. The direct cost of using a service could be prohibitive.

*“With difficulty we make money to have food to eat, so from where will we get money for medicines? I cannot pay money for my health and receive treatment in private health care facilities. I prefer to go to government hospitals which are free.” (ID#2, F, 58yr)*

*“Costs are very high at the private tertiary hospital, and we need to take loans to be treated there.” (ID#1, M, 58yrs)*

Travel costs were a consideration, and so was the time away from home and the demands of domestic work. Therefore, participants often preferred to seek initial treatment from services that were cheaper and close to their place of residence, often pharmacies or small clinics.

*“I prefer being treated closer to home rather than travelling long distances with additional costs.” (ID#2, F, 58yrs)*

*“Smaller clinics locally are cheaper, and we can receive treatment for even INR 100 [USD1.27].” (ID#1, M, 58yrs)*

Patients noted the potential financial impact of illness and prioritized low-cost relief of symptoms, allowing a return to work or daily routines.

*“My salary was cut, as I missed work for more than a month.” (ID#10, M, 50yrs)*

*“If I’m unable to do my daily work and symptoms are worsening, I quickly get something from the pharmacy, so I can resume my work.” (ID#4, F, 63yrs)*

Many cases described injections as a desirable form of rapid treatment at local clinics, but one case noted side effects from receiving injections which could interfere with return to work, and therefore preferred medicines from pharmacies.

*“If I go to the pharmacy and get some medicines, I can get back to my work without feeling tired. Injections at local clinics cause more tiredness, and I need to sleep longer, which makes it difficult to get back to the daily work.” (ID#5, F, 32yrs)*

The availability of doctors at a convenient time outside of working hours was also a consideration to minimize loss of earnings.

*“Local clinics are preferred … the doctor is available in the evenings.” (ID#12, F, 60yrs)*

Decisions on the choice of provider, in the initial days of fever, were also based on convenience.

*“We first go to the private clinic, as it is close by. They usually give an injection and some medicines. They give us some idea of what the situation is and then we can plan better. If symptoms are not improving, then we could go to a higher centre like the private secondary hospital close by. I don’t usually go to this private hospital, as I need to go from one counter to another and I don’t know how to read the numbers. In the government hospital, I only need to go into one room to see the doctor and just one area for the medicines.” (ID#2, F, 58yr)*

Participants assessed possible healthcare providers in terms of the likelihood of getting effective and cheap medical care while being treated with respect. Supportive and trusted medical staff could be integral to this. Prior experience and the experience of others were important sources of information in selecting a healthcare provider.

*“We go to where we are familiar, where we know we will get better, with similar experiences in the past.” (ID#13, M, 68yrs)*

Personal relationships or connections with known individual staff members were important factors in selecting health care providers.

*“My son’s boss knew someone in the nursing section at the government hospital. He made a phone call for us and with this they really took good care of me.” (ID#2, F, 58yrs)*

*“My daughter’s husband has relatives working at the private tertiary hospital, so we went there.” (ID#4, F, 63yr)*

However, participants also reported changes between several healthcare facilities over a short period of time with little success, after receiving often unspecific advice as to where to go next:

*“The first clinic said the fever is too high, we can’t treat here, so I went to the next clinic. Every hospital we went to confirm a high-grade fever but wouldn’t take further action. They continued to tell us to go to a larger hospital.” (ID#8, F, 19yrs)*

#### Disease Factors.

Fever was considered common in this area and not serious in adults. Seriousness was associated with persistence of fever, development of additional symptoms or significant interference with daily activities, including ability to eat or drink. Only then was treatment from a qualified healthcare professional and diagnostic procedures sought.

*“I took initially medicines from the local clinic, but the following day I suddenly felt unwell with chills, and the fever was also restarting. My father-in-law said that I needed to go to the hospital and ask why the fever is recurring. There they would test and find out what kind of fever it is.” (ID#9, F, 23yrs)*

There was some familiarity with malaria and dengue fever as causes for fever. However, scrub typhus as a disease was unknown to most respondents and many local health care practitioners. Some local terms of severe fever matching symptoms of scrub typhus included “insect bite fever”, “brain fever” or “pneumonia” (using the English term).

*“They did a test at the clinic with a glass and informed me that it wasn’t due to any specific type of fever. So, if I got the insect bite fever, why wouldn't they know about it?” (ID#4, F, 63yrs)*

#### Patient Factors.

Patient factors influencing the choice of healthcare included age, sex and social support. Fever in children and pregnant women was regarded as more serious, usually prompting a visit to a qualified professional or private hospital within one or two days of the start of the fever.

*“When my son was a child and was having fever, we took him directly to the private tertiary hospital.” (ID#11, F, 68yrs)*

One participant was ill during her pregnancy and within a day of her fever, was advised by family and friends to see a specialist,

*“…a lady doctor who sees especially pregnant women, she is an MBBS [qualified] doctor.” (ID#8, F, 19yrs)*

Some participants referred to the need for social support. They described decision making about health care being constrained by domestic duties. They further quoted lack of general support during hospitalisations (most hospitals require a relative to be present at all times). Therefore, the choice of providers was also influenced by the kind of support available.

*“I was told to go immediately that evening to the tertiary private hospital in the city, but I waited one night, as no one was there to care for my other children at home. We only went the next day.” (ID#5, F, 32yrs)*

*“I went to a clinic close by, as they could give an injection and some medicines to be taken at home. If admitted [to a hospital], there is no one who can stay with me at the hospital.” (ID#2, F, 58yrs)*

Some women expressed the difficulty of travelling alone to distant health facilities.

*“I must wait for my husband to go anywhere further than the local clinic or local government hospital.” (ID#7, F, 28yrs)*

## Discussion

The diagnosis and treatment of scrub typhus have a large economic impact on patients in this setting, both in terms of direct and indirect costs. In settings that are highly endemic for scrub typhus, the economic burden of scrub typhus is likely to be large, given a high incidence of scrub typhus-related hospitalisations [15]. The qualitative study highlighted the difficulties faced by affected households in obtaining treatment for scrub typhus in a highly fragmented and (at the primary level) often unregulated health care system. Decisions on choice of providers were largely driven by costs, convenience, disease perception and personal connections.

Mean overall costs for illness in our study were USD 189, with mean overall costs in severe cases exceeding USD 1300. Similar orders of magnitude for direct costs have been reported for other fevers in India, most prominently dengue fever, with severe cases in pediatric and adult patients having median direct costs of USD 933 and USD 720 in 2018 prices [25]. In a study comparing the cost of illness for dengue and chikungunya fever in Gujarat, the mean costs were three times lower for both infections compared to scrub typhus reported here. The Gujarat study offered no breakdown by disease severity, and mild cases may have predominated [26]. Unlike dengue, severe disease in scrub typhus is potentially preventable by early use of cheap antibiotics such as doxycycline and azithromycin, highlighting the role of treatment delays in contributing to the financial burden associated with the disease. Obtaining a timely presumptive or definite diagnosis of scrub typhus and treatment is however difficult in our study setting, marked by a highly fragmented healthcare landscape in a rural setting, that often led to the use of multiple unconnected healthcare providers. Successive inadequate and incomplete treatment courses for fevers has been observed in other parts of the region [27].

A total of 69 cases were hospitalised with mean overall costs of approximately USD 700, which was USD 443 more than the cost of ambulatory cases after adjusting for potential confounders (Table 4). The daily wage for an individual in this area ranges from approximately USD 6 to USD 10. The mean overall cost for an admission of a severe case around USD 1300 or a hospitalisation around USD 700 can cause a considerable debt burden on households. In our study, catastrophic health expenditure exceeding 10% and 25% thresholds were found to affect 15% and 10% of cases, respectively, and was very common in severe infections and in the case of hospitalisation. Only one participant had private insurance which could be used for the treatment costs. Hospitalisations were common even amongst non-severe cases, which might have been avoided by better diagnostics. The use of rapid diagnostics tests at the primary and secondary level may prevent potentially unnecessary hospitalisations, as those with confirmed scrub typhus and in a stable condition could be sent home on antibiotics. In those with more severe infections, having a confirmed rapid test could avoid unnecessary costs for the diagnostic work up for other fevers.

In our study, both overall and direct costs of illness were found to be twice as high in males compared to females. This effect was reduced after adjusting for disease severity (males may be more susceptible to severe infection [15]). Some difference, however, remained, suggesting a tendency to invest more in the health of males than in that of females. This finding aligns with studies from India that document comparable gender disparities in inpatient healthcare expenditure across socioeconomic strata, with women receiving lower levels of health spending than men for conditions requiring inpatient care [28,29].

In the qualitative study, a recurring theme was the preference for immediate treatment close to home without additional diagnostics, largely to avoid high costs, including the opportunity costs from missed work and the need for social support. This preference is not unreasonable, given that most fevers (including fever due to scrub typhus [15]) present as self-limiting infections that do not require treatment. The largely unregulated system of untrained practitioners and pharmacies dispensing medicines without prescriptions enables this option to remain an important first level of care in fevers across India [30,31] and in other settings as well [32]. However, there is an urgent need to better understand which medicines are dispensed at the primary level, as inappropriate treatments, such as unnecessary corticosteroids or antibiotics, pose substantial risks of harm [32]. Perhaps surprisingly, very few cases opted to receive no treatment at all, even for mild, short-duration fevers. Abhijet et al. reported similar findings from their study in Udaipur, noting that although poorer individuals could not afford expensive treatments, they often insisted on obtaining some form of low-cost, frequently unnecessary treatment, which gave them a sense of agency and control over their health [33]. The same may apply to the use of traditional healers, which was rare in the quantitative study (Table 2), but emerged as a common theme in the qualitative study, suggesting considerable under-reporting of this treatment option in the former.

We found that higher level treatment facilities were usually sought only when the condition was clearly serious, bearing in mind the financial and care burden on the whole family. Only if the case involved a child or a pregnant woman was there a demand for treatment by a qualified healthcare provider and for further diagnostics, even in cases of mild fever.

In severe cases, private hospitals were visited more often than government hospitals. This finding is supported by qualitative study findings, which suggest that individuals have greater trust in private hospitals than government facilities. The importance of having a personal connection with hospital staff was considered even greater in government hospitals than in private hospitals. Although treatment in the government sector is free or very inexpensive, the motivation of staff is perceived to be low.

An important limitation of this study is its generalizability, as this cohort was in rural villages with a known high endemicity for scrub typhus. The cost of illness due to scrub typhus may also depend on the quality of the health care system, in particular on early case recognition and treatment to prevent severe illness. Information related to the disabilities associated with the illness after treatment, or in the case of death, the long-term impact on the quality of life and social status of affected families have not been systematically assessed here. Data on one death in a male case occurring in the main study [15] could not be collected as the family relocated following the death, possibly due to economic pressure. The true economic burden on the individual is often difficult to quantify in monetary terms. When a male or female household head is ill, the entire household is affected, which would indirectly impact the health and well-being of children and other family members. Loss of time for education in college students and school children poses similar challenges. We attempted to account for this by assuming a loss of income as indirect costs for students, but the validity of these assumptions remains uncertain. The qualitative study was restricted to a relatively small number of in-depth interviews in severe cases. Focusing on severe infection may have biased the qualitative findings towards cases with a particularly negative experience of the health care system.

To conclude, this study highlights the considerable costs of scrub typhus infection in a highly endemic setting in South India. Preventing severe infection and hospitalisation by early use of effective antibiotics has large potential for reducing the economic burden of scrub typhus. However, this study highlighted several barriers to achieving this. The need to create awareness regarding this common disease among the unregulated and untrained practitioners at the primary care level is evident but given the informal nature of the sector may be difficult to enforce. Among health care providers that can be reached, awareness of the disease may be strengthened by aiming at the inclusion of antibiotics effective against scrub typhus into existing approaches to presumptive treatment. Treatment seeking behaviour in this population was generally based on rational and pragmatic considerations that may be open to public awareness campaigns in a setting where the disease is still largely unknown. A better understanding of health care seeking behaviour and health systems in addition to improving diagnostics and treatment is crucial in order to reduce the economic burden of scrub typhus.
