# Correction: Cost of illness of scrub typhus in South India – a population-based, mixed-methods study

**DOI:** 10.1371/journal.pntd.0014712

**Published:** 2026-09-10

**Authors:** Carol Devamani, Adam Biran, Koya Ariyoshi, K.R. John, Ian Ross, Yoshinao Kubo, Daniel Chandramohan, Kundavaram Paul Prabhakar Abhilash, Wolf-Peter Schmidt

An additional affiliation is missing for the first and sixth author. Carol Devamani and Yoshinao Kubo are also affiliated with the Graduate School of Biomedical Sciences, Nagasaki University, Nagasaki, Japan.
